# Granulocyte Colony-Stimulating Factor Ameliorates Endothelial Activation and Thrombotic Diathesis Biomarkers in a Murine Model of Hind Limb Ischemia

**DOI:** 10.3390/biomedicines10092303

**Published:** 2022-09-16

**Authors:** Angeliki Valatsou, Panagiotis Theofilis, Spyridon Simantiris, Georgia Vogiatzi, Alexandros Briasoulis, Marios Sagris, Evangelos Oikonomou, Alexios S. Antonopoulos, Alkistis Pantopoulou, Narjes Nasiri-Ansari, Elizabeth Fragopoulou, Despoina Perrea, Konstantinos Tsioufis, Dimitris Tousoulis

**Affiliations:** 1 First Department of Cardiology, “Hippokration” General Hospital, University of Athens Medical School, 11527 Athens, Greece; 2 Third Department of Cardiology, Thoracic Diseases General Hospital “Sotiria”, University of Athens Medical School, 11527 Athens, Greece; 3 Division of Cardiovascular Medicine, University of Iowa Hospitals and Clinics, Iowa City, IA 52242, USA; 4 Laboratory of Experimental Surgery and Surgical Research “N.S. Christeas”, University of Athens Medical School, 11527 Athens, Greece

**Keywords:** granulocyte colony-stimulating factor, inflammation, endothelium, adhesion molecule, plasminogen activator inhibitor-1, limb ischemia

## Abstract

Novel therapies in peripheral arterial disease, such as granulocyte colony-stimulating factor (GCSF) administration, might result in anti-atherosclerotic effects. In this study, we used 10-week-old male ApoE^−/−^ mice, which were fed an atherosclerosis-inducing diet for four weeks. At the end of the four weeks, hind limb ischemia was induced through left femoral artery ligation, the atherosclerosis-inducing diet was discontinued, and a normal diet was initiated. Mice were then randomized into a control group (intramuscular 0.4 mL normal saline 0.9% for 7 days) and a group in which GCSF was administrated intramuscularly in the left hind limb for 7 days (100 mg/kg). In the GCSF group, but not in the control group, we observed significant reductions in the soluble adhesion molecules (vascular cell adhesion molecule-1 (sVCAM-1) and intercellular adhesion molecule-1 (sICAM-1)), sE-Selectin, and plasminogen activator inhibitor (PAI)-1 when they were measured through ELISA on the 1st and the 28th days after hind limb ischemia induction. Therefore, GCSF administration in an atherosclerotic mouse model of hind limb ischemia led to decreases in the biomarkers associated with endothelial activation and thrombosis. These findings warrant further validation in future preclinical studies.

## 1. Introduction

Atherosclerotic diseases remain a leading cause of morbidity and mortality worldwide [1,2] despite recent advances in their management. Although coronary and carotid artery disease are frequently at the epicenter of scientific investigation, the prevalence of lower extremity artery disease, commonly referred as peripheral artery disease (PAD), is not negligible, increasing over time and nowadays exceeding 230 million adults worldwide [3]. Atherosclerosis is also considered the main pathophysiologic substrate of PAD and shares common risk factors with disease in other vascular beds. Diabetes mellitus (DM) and smoking represent the two strongest PAD risk factors [4,5,6]. Patients with PAD are at a higher risk of mortality, myocardial infarction (MI), and stroke compared to patients without PAD [7,8].

PAD treatment mostly focuses on antithrombotic therapy and risk factor modification, with treatment options including statins, smoking cessation, hypertension and DM control, and lifestyle changes or interventional treatment [9,10]. Newer therapies have also been suggested, such as granulocyte colony-stimulating factor (GCSF) and granulocyte–macrophage colony-stimulating factor (GM-CSF) [11,12,13]. GCSF is a cytokine that mobilizes granulocytes and stem cells from the bone marrow such as CD34+ endothelial progenitor cells (EPCs) [14]. EPCs can migrate to ischemic regions and mediate arteriogenesis, leading to more sufficient peripheral tissue perfusion [15]. GCSF treatment increased collateral flow and reduced infarct volume in experimental brain and myocardial ischemia models [16,17,18,19,20]. A recent preclinical study also showed that subcutaneous and intracoronary GCSF treatment improved the severity of distal stenoses and decreased the infarct size and also ameliorated ventricular remodeling and function due to pro-angiogenic and anti-apoptotic actions [21]. In a randomized study of patients with stable coronary artery disease, the pegylated GCSF analogue pegfilgrastim improved collateral function and anginal symptomatology compared to a placebo [22].

GCSF administration and GCSF-mobilized autologous peripheral blood monocyte transplantation have been assessed in both experimental models and in clinical trials of PAD. Both have been associated with an improvement in the symptoms, ankle brachial index values, and clinical severity stage in PAD patients with a favorable side effect profile [23,24,25,26]. In a recent study of patients with chronic limb ischemia, the GCSF analogue filgrastim, in combination with an infra-geniculate programmed compression pump, resulted in improved fibrinolysis and angiogenesis compared to the programmed compression pump alone [27]. The upcoming GPAD-3 study is expected to provide further evidence for the clinical efficacy of GM-CSF in this group of patients [13]. Other than affecting angiogenesis, GCSF’s anti-atherosclerotic effect may extend to other deleterious processes, such as endothelial dysfunction and thrombosis. Therefore, in the present study, we assessed the effect of GCSF administration on biomarkers of endothelial activation and thrombosis in a murine model of hind limb ischemia.

## 2. Materials and Methods

### 2.1. Study Design

The study population consisted of 20 male ApoE^−/−^ mice aged 10 weeks and weighing 25–30 g. Mice were obtained from the Jackson Laboratory (Bar Harbor, ME, USA). The number of experimental animals selected was based on the existing literature [28], and after appropriate statistical processing, it is considered to be the minimum possible number to make an evaluation of the results feasible (power level 90%). The mice were fed an atherosclerosis-inducing diet (2% rich in cholesterol) for 4 weeks prior to the experiment. All of the mice lived in an environment with constant temperature (22–25 °C) and humidity (60 ± 5%) conditions and with a 12 h light-dark cycle and free access to food and water throughout the experimental procedures. At the age of 14 weeks, left limb ischemia was performed via the double ligation and resection of the left common femoral artery. Immediately after surgery, the atherosclerotic diet was replaced with a normal diet (not high in fat).

Mice were then randomized into a control group (intramuscular 0.4 ml normal saline 0.9% for 7 days) or a group in which GCSF was administrated intramuscularly in the left hind limb for 7 days (100 mg/kg). Blood was drawn from the ophthalmic artery the day after hind limb ischemia induction (1st day) and on the 28th day. Soluble adhesion molecules ((vascular cell adhesion molecule-1 (sVCAM-1), intercellular adhesion molecule-1(sICAM-1), and sE-Selectin), and plasminogen activator inhibitor (PAI)-1 were measured through enzyme-linked immunosorbent assay (ELISA), and the differences between the two groups at the examined time points were assessed. On the 28th day after ligation, euthanasia was performed through the administration of high doses of anesthesia (ketamine hydrochloride).

### 2.2. Limb Ischemia

Ischemia of the left mouse limb was performed under aseptic conditions using the following procedure: under anesthesia, through the intramuscular administration of ketamine hydrochloride (100 mg/kg) and xylazine (10 mg/kg) as well as ether per os, each animal was placed on the operating table in supine position, with the left limb being extended and stabilized. Topical antiseptic was applied (povidone iodine with ethyl alcohol), and a vertical incision was made in the left groin. The left common femoral artery, identified by its light pink color and the presence of a pulse, was separated from the common femoral vein, and the femoral nerve and was ligated at both ends with a prolene 6.0 nylon suture. Subsequently, the section between the ligation sites was removed, thus allowing the formation of a collateral network, and the skin incision was closed with three absorbable 5.0 sutures. Finally, each mouse was placed in its cage and was closely monitored until the anesthetic effects elapsed.

### 2.3. Laser Doppler Perfusion Imaging

Immediately after the performance of femoral artery ligation, we scanned the respective hind limbs with a laser Doppler flowmeter (Perimed, Järfälla, Sweden) to document the successful occlusion. The mice were stabilized under the flowmeter after brief anesthesia using ether inhalation, and three consecutive scans that were 3 min in duration were performed. Qualitative evaluation of the images revealed successful occlusion in the entire study population.

### 2.4. Enzyme-Linked Immunosorbent Assay

The concentration of adhesion molecules was assessed from the mouse serum using ELISA. For ELISA, we used the MOUSE CARDIOVASCULAR DISEASE (CVD) PANEL 1 (LINCOplex KIT 96 Well Plate Assay (Cat. MCVD1-77AK), Fluorescent-labeled microsphere beads. Bio-Rad Laboratories, Inc.^®^, Hercules, CA, USA, (Bio-Plex ™)). This is a kit that includes microspheres that are immobilized by antibodies and is used for the simultaneous quantification of many substances in any combination. The adhesion molecules were measured in sera collected on the first day after ischemia, before the agents were administered, as well as on the 28th day, before performing euthanasia. The samples were centrifuged at 1500 rpm for 15 min. The sera were isolated and maintained at −20 °C in ethylenediamine tetraacetic acid (EDTA) solution until their use for ELISA performance. ELISA results were expressed in ng/mL and as the median fluorescence intensity (MFI). Data storage and evaluation were conducted using the SPSS software (version 25.0, SPSS Inc., Chicago, IL, USA).

### 2.5. Study Ethics

The present study was performed in the Experimental Surgery and Surgical Research Laboratory “N.S. Christeas”, National and Kapodistrian University of Athens. Written approval to conduct this experimental protocol was obtained from the Ethics Committee of the General Hospital of Athens “Hippokration”, and the protocol received certification from the Medical School of the National and Kapodistrian University of Athens (protocol number: 125/2016). In addition, a permit approving the experiments on the mice was obtained by the Veterinary Service of Athens.

### 2.6. Statistical Analysis

The mean ± standard error of the mean of the examined biomarkers was calculated in each intervention group on days 1 and 28. We evaluated the distribution of the continuous variables with the Kolmogorov–Smirnov test, and it was found that they do not follow the normal distribution. Hence, the application of a non-parametric test (Mann–Whitney U) for a comparison of the between-group differences in the continuous variables was considered necessary. The magnitude of the change in the biomarkers in each group was assessed using the Wilcoxon signed-rank test. All of the reported *p* values were based on two-sided hypotheses. A *p* value of <0.05 was considered statistically significant. All statistical calculations were performed using SPSS software (version 25.0; SPSS Inc., Chicago, IL, USA).

## 3. Results

### 3.1. Granulocyte Colony-Stimulating Factor Administration Reduces Endothelial Activation

**Figure 1:**
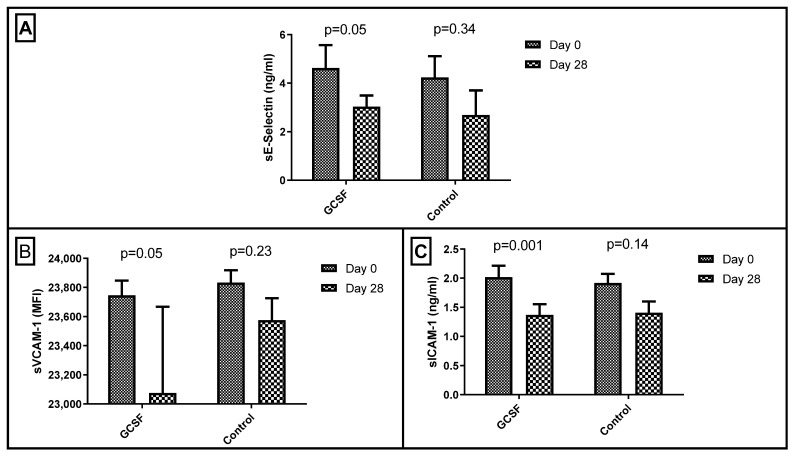
Differences in (**A**) soluble (s) E-Selectin, (**B**) s vascular cell adhesion molecule (VCAM)-1, and (**C**) s intercellular adhesion molecular (ICAM)-1 on 1st and 28th days after limb ischemia induction in the control and granulocyte colony-stimulating factor (GCSF) group.

On the first day of the experiment, the endothelial activation biomarker values did not differ significantly between the control and the GCSF group (sE-selectin: 4.24 ± 0.88 ng/mL vs. 4.63 ± 0.95 ng/mL with *p* = 0.77, sVCAM-1: 23,384 ± 84 MFI vs. 23,746 ± 101 MFI with *p* = 0.51, and sICAM-1: 1.92 ± 0.16 ng/mL vs. 2.02 ± 0.2 ng/mL with *p* = 0.70). In the control group, no significant difference was identified in the biomarker levels between the 1st and the 28th days (*p* > 0.14 for all). On the other hand, in the GCSF group, sE-Selectin, sICAM-1, and sVCAM-1 were significantly reduced (*p* = 0.04, 0.01, and 0.04, respectively, Table 1 and Figure 1).

### 3.2. Granulocyte Colony-Stimulating Factor Administration Attenuates Thrombotic Diathesis

**Figure 2:**
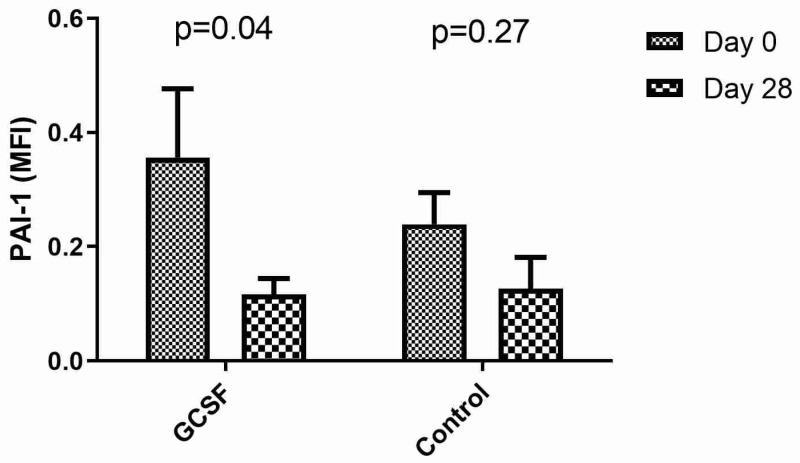
Differences in plasminogen activator inhibitor (PAI)-1 on 1st and 28th days after limb ischemia induction in the control and granulocyte colony-stimulating factor (GCSF) groups.

On the first day of the experiment, the biomarker values did not differ significantly between the control and the GCSF group (PAI-1: 0.239 ± 0.055 MFI vs. 0.356 ± 0.121 MFI with *p* = 0.39). In the control group, no significant differences were identified in the biomarker levels between the 1st and the 28th days (*p* = 0.27). On the other hand, in the GCSF group, PAI-1 levels were significantly reduced (*p* = 0.04, Figure 2).

## 4. Discussion

PAD still represents a significant cause of morbidity, and current management techniques, including invasive treatment with either percutaneous or surgical revascularization, is often ineffective in preventing limb amputation [29]. GCSF administration has been examined in many preclinical and clinical studies of peripheral arterial disease as a potent angiogenic factor, and it has been associated with favorable outcomes [12,23,24,25,26,30,31]. The aim of this study was tο assess the effects of GCSF on endothelial activation and thrombosis through the use of relevant biomarkers. We have demonstrated that GCSF administration after hind limb ischemia reduces the levels of biomarkers associated with endothelial activation, such as adhesion molecules sE-Selectin, sICAM-1, and sVCAM-1, in an atherosclerosis-prone murine model. Moreover, we noted a significant decline in the levels of PAI-1 after treatment with GCSF, which is indicative of attenuated thrombotic processes. Further validation of our results may shed light on the potentially beneficial role of GCSF as an anti-atherosclerotic agent through influencing critical processes such as endothelial dysfunction and thrombosis.

The imbalance between the vasodilators and vasoconstrictors that are generated by endothelial cells in the presence of endothelial dysfunction results in an atheroprone phenotype that includes vasoconstriction and leukocyte mobilization. Endothelial nitric oxide (NO) synthase expression and activity are diminished, which results in decreased NO production and availability. This is typically the first mediator of endothelial dysfunction. Increased oxidative stress causes the creation of superoxide to replace NO, a process known as NO uncoupling, which has detrimental side effects such the development of the pro-oxidant peroxynitrite, which supports mitochondrial and endothelial cell failure [32,33]. Inflammation, a cause and an outcome of endothelial dysfunction [34], is also crucial in the development of vascular damage, as it may mediate platelet activation and coagulation [35,36], potentially leading to major adverse cardiovascular events.

Adhesion molecules are considered essential in endothelial activation, as they aid monocyte and leukocyte adherence and rolling. As biomarkers, E-Selectin and ICAM-1 have been associated with coronary and carotid artery disease, independently of other cardiovascular risk factors [37]. Additionally, sICAM-1 could be a prognostic marker in PAD since disease progression to myocardial infarction or peripheral revascularization was noted in those with higher circulating levels [38]. Moreover, VCAM-1 may be related to the extent of coronary arterial lesions [39] as well as to PAD diagnosis [40]. We have shown a reduction in the concentration of circulating soluble adhesion molecules with GCSF administration, which could be indicative of ameliorated endothelial function.

Other than the documented action of GCSF on arteriogenesis [41], the effect of GCSF administration on the endothelium has been assessed by many studies offering conflicting evidence. There are other studies supporting the favorable effects of GCSF on the endothelium. In the study of Ikonomidis et al., GCSF administration in 60 women with stage I breast cancer after adjuvant chemotherapy led to a greater increase in the flow-mediated dilatation (FMD) of the brachial artery compared to a placebo. This effect was attributed to an increase in anti-inflammatory cytokines (IL-10) and a simultaneous decrease in pro-inflammatory cytokines (TNF-α) [42]. Subcutaneous injection of GCSF for 5 days after sirolimus-eluting stent (SES) implantation was compared to a placebo and was associated with attenuated vasoconstriction 15 mm distal to the stent after acetylcholine infusion in an angiography follow up. In this way, GCSF reduced the burden of endothelial dysfunction related to SES implantation [43]. On the other hand, in a study of 15 healthy donors, subcutaneous administration of GCSF was associated with an increase in sE-Selectin levels and steady sICAM-1 levels [44]. Similarly, an increase in sE-selectin, sICAM-1, and sVCAM-1 was observed in 10 healthy subjects between the first and the fifth days of GCSF administration compared to the controls [45]. At the cellular level, human umbilical vein endothelial cells exposed to GCSF express significantly higher levels of adhesion molecules, E-selectin, VCAM-1, and ICAM-1 [46].

The disruption of the thrombosis–fibrinolysis balance is another determinant of atherosclerosis [47] since it may mediate fibrin deposition, extracellular matrix turnover, and cell migration. Fibrinolysis is regulated by plasminogen conversion to plasmin by plasminogen activators (urinary- and tissue-type). However, PAI-1 may oppose fibrinolysis by inhibiting the action of plasminogen activators [48], thus aiding thrombosis and, consequently, atherosclerosis progression [49]. Increasing PAI-1 levels have been found to be related to a higher prevalence of PAD [50], while PAD revascularization resulted in sustained PAI-1 concentration decrease [51]. Moreover, pharmacological targeting of PAI-1 has been attempted, with anti-atherosclerotic effects [52]. Interestingly, GCSF administration led to significantly lower PAI-1 levels compared to the control group, highlighting a putative antithrombotic effect of GCSF. To our knowledge, this is the first study to report a lowering of PAI-1 through the use of GCSF. Previous case reports have pointed to potential hypercoagulability following GCSF utilization [53,54]. However, GCSF administration in patients with acute myocardial infarction, which resembles our modeled acute limb ischemia, did not alter the coagulation and platelet activation parameters [55]. This speculated antithrombotic effect of GCSF deserves further validation in future preclinical studies of modeled acute cardiovascular events as well as in in vitro studies of platelet activation and coagulation.

## 5. Limitations

Our study has a few limitations. To begin with, we did not evaluate the use of GCSF in experimental models of a normal diet or without femoral artery ligation; thus, the validity of our findings in this regard should be interpreted cautiously. Moreover, the selected parameters of interest were only examined on days 1 and 28; therefore, the lack of serial measurements does not allow for a more precise assessment of their dynamics.

## 6. Conclusions

The intramuscular administration of granulocyte colony-stimulating factor in an atherosclerosis-prone mouse model of hind limb ischemia ameliorated the indices of endothelial dysfunction and thrombosis. These putative endothelial protective and antithrombotic effects merit further research to appropriately determine the future role of this intervention in peripheral arterial disease and in atherosclerosis in general.

## Figures and Tables

**Table 1:** Differences in soluble (s) adhesion molecules and plasminogen activator inhibitor-1 (PAI-1) between days 0 and 28 in the control and GCSF group.

	Control	*p*	GCSF	*p*
sE-Selectin (Day 1), ng/mL	4.24 ± 0.88	0.34	4.63 ± 0.95	0.04
sE-Selectin (Day 28), ng/mL	2.69 ± 1.01		3.03 ± 0.46	
sICAM-1 (Day 1), ng/mL	1.92 ± 0.16	0.14	2.02 ± 0.20	0.01
sICAM-1 (Day 28), ng/mL	1.41 ± 0.19		1.37 ± 0.18	
sVCAM-1 (Day 1), MFI	23,834 ± 84	0.23	23,746 ± 101	0.04
sVCAM-1 (Day 28), MFI	23,575 ± 151		23,075 ± 592	
PAI-1 (Day 1), MFI	0.239 ± 0.055	0.27	0.356 ± 0.121	0.04
PAI-1 (Day 28), MFI	0.126 ± 0.055		0.122 ± 0.026	

Data are expressed as mean ± standard error of the mean. Differences between days 1 and 28 were assessed with the Wilcoxon signed-rank test. GCSF: granulocyte colony-stimulating factor, ICAM-1: intercellular adhesion molecule-1, VCAM-1: vascular cell adhesion molecule-1.

## Data Availability

The data supporting this research are available upon reasonable request from the corresponding author.
